# Minimally invasive surgery treatment for the patients with spontaneous supratentorial intracerebral hemorrhage (MISTICH): protocol of a multi-center randomized controlled trial

**DOI:** 10.1186/s12883-014-0206-z

**Published:** 2014-10-10

**Authors:** Jun Zheng, Hao Li, Rui Guo, Sen Lin, Xin Hu, Wei Dong, Lu Ma, Yuan Fang, Anqi Xiao, Ming Liu, Chao You

**Affiliations:** Department of Neurosurgery, West China Hospital, Sichuan University, Chengdu, Sichuan 610041 China; Department of Neurology, West China Hospital, Sichuan University, Chengdu, Sichuan 610041 China

**Keywords:** Intracerebral hemorrhage, Minimally invasive surgical treatment, Craniotomy, Neuroendoscope, Stereotactic aspiration

## Abstract

**Background:**

The choice of surgical or conservative treatment for patients with spontaneous intracerebral hemorrhage is controversial. Some minimally invasive treatments have been applied to hematoma evacuation and could improve prognosis to some extent. Up to now, studies on minimally invasive surgery for patients with spontaneous intracerebral hemorrhage are still insufficient.

**Design:**

The MISTICH is a multi-center, prospective, randomized, assessor-blinded, parallel group, controlled clinical trial. 2448 eligible patients will be assigned to neuroendoscopy group, stereotactic aspiration group and craniotomy group randomly. Patients will receive the corresponding surgery based on the result of randomization. Surgeries will be performed by well-trained surgeons and standard medical treatment will be given to all patients. Patients will be followed up at 7 days, 30 days, and 6 months. The primary outcome of this study is unfavorable outcome at 6 months. Secondary outcomes include: mortality at 30 days and 6 months after surgery; neurological functional status of 6 months after surgery; complications including rebleeding, ischemic stroke and intracranial infection; days of hospitalization.

**Discussion:**

The MISTICH trial is a randomized controlled trial designed to determine whether minimally invasive surgeries could improve the prognosis for patients with spontaneous intracerebral hemorrhage compared with craniotomy. (ChiCTR-TRC-12002026. Registered 23 March 2012).

## Background

Spontaneous intracerebral hemorrhage (sICH) is the most devastate kind of all stokes. It was estimated that sICH affects over 1 million people worldwide every year [1,2]. The case fatality rate of sICH at 30 days is about 30%-55%, and only 12%-39% of the survivors could live independently after 6 months [3-5].

The treatment of sICH is still controversial among neurosurgeons and neurologists. The focus of debate is whether evacuation of hematoma would be able to improve the prognosis of patients. Previous studies showed that the removal of hematoma might reduce nervous tissue damage, possibly by relieving local ischemia and removing noxious chemicals [6-8]. Several studies aiming to explore the efficacy of surgery for patients with sICH have been carried out but showed different results. In 1961, Mickissock and colleagues published the first prospective randomized controlled trial. Their results showed that patients treated surgically had worse outcome than the patients in the conservative group [9]. Another influential study is the STICH series trials, which concluded that there was no overall benefit of early surgery for patients with supratentorial sICH compared with conservative treatment [10,11]. Some researchers attributed this to additional surgical traumatization. In order to reduce surgical traumatization, some minimally invasive techniques have been used in hematoma evacuation. These minimally invasive techniques include stereotactic aspiration and neuroendoscope assisted surgery. The stereotactic aspiration guided by CT was successfully applied for hematoma evacuation by Backlund and collogues at 1978, and this minimally invasive surgery was further improved by some scholars [12]. It was reported that the stereotactic aspiration combined with fibrinolytic drugs could be more effective in hematoma evacuation than aspiration alone [13]. Another category of minimally invasive technique is neuroendoscope. Auer et al. reported that neuroendoscope could be applied to hematoma evacuation, but a subsequent randomized controlled trial showed that the outcome of surgical patients with putaminal or thalamic hemorrhage was not better than medical treatment [14,15]. Recently, a lot of studies exploring the efficacy of minimally invasive surgery (MIS) compared with conservative craniotomy or medical treatment were carried out, but none of them provided sufficient evidence regarding the choice of treatment [16-20]. A further meta-analysis showed that patients with supratentorial intracerebral hemorrhage might benefit more from MIS than other treatment options [21]. Though MIS seems less invasive than the traditional craniotomy, it was reported that the incidence of some complications (e.g. rebleeding and infection) was higher than craniotomy. In addition, with assistance of neuronavigation and operative microscope, hematoma evacuation by craniotomy could also be minimally invasive [22,23]. Until now, there is no randomized controlled trial comparing the efficacy of neuroendoscopy, stereotactic aspiration and craniotomy in patients with spontaneous intracerebral hemorrhage except a small-scale trial by Cho and colleges. Thus, a large-scale clinical trial is necessary to provide robust evidence for clinical practice by assessing the safety and efficacy of different surgical methods including neuroendoscopy, CT-stereotactic aspiration and neuronavigation-assisted craniotomy for sICH. Here we designed a randomized, assessor-blinded, parallel-group, controlled, multi-center clinical study termed minimally invasive surgery treatment for patients with spontaneous supratentorial intracerebral hemorrhage (MISTICH).

## Design

**Figure 1:**
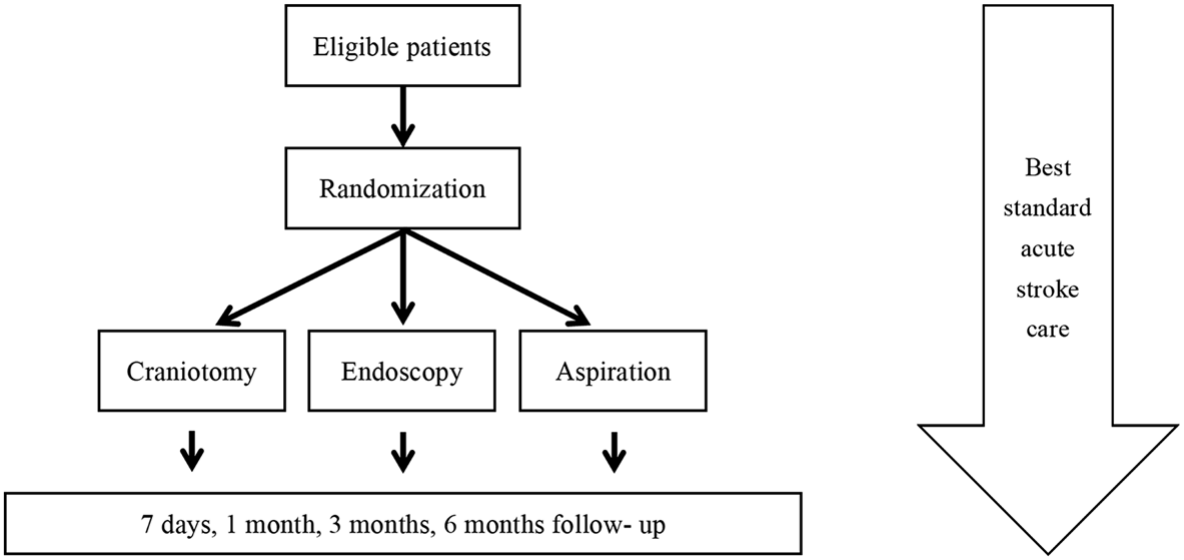
**Flow diagram of the MISTICH study.**

The MISTICH is a multi-center, prospective, randomized, assessor-blinded, parallel group, controlled clinical trial. The overall flow of MISTICH is showed in Figure 1. A total of 20 centers from around China are included. The centers were eligible if they: have the ability to carry out all kinds of minimally invasive surgery of this trial; demonstrate adequate trial experience; have previous adherence to trial guidelines with high follow-up rates. All participating hospital sites are to receive approval from the relevant ethics committee before initiation of the study. Until now, 20 centers are currently involved with this study and all of the centers have already received ethical approval. This study has been registered in the Chinese Clinical Trial Registry (ChiCTR-TRC-12002026). All the patients or their legal surrogate will be fully informed and informed consent will be signed in this trial.

### Study objective

The primary objective is to evaluate different minimally invasive techniques including neuronavigation assist craniotomy, neuroendoscopy, and CT-stereotactic aspiration on their unfavorable outcomes at 6 months in patients with spontaneous intracerebral hemorrhage. The secondary objective is to investigate the safety of different minimally invasive techniques. Another objective is to evaluate the separate effects of treatment on death and dependency through physical function, complications, and days of hospitalization.

### Patient population

#### Inclusion criteria

1. Age between 15 and 75 years;
2. Diagnosed with acute spontaneous supratentotial intracranial hemorrhage (Meets U.S. adults spontaneous intracerebral hemorrhage treatment guidelines (2010) diagnostic criteria)
3. Within 48 hours post ictus;
4. With Glasgow Coma Scale (GCS) between 5 and 12;
5. CT scan showed hematoma volume is 30 ml or above;
6. Agree to participate in this clinic trial and sign the informed consent.

#### Exclusion criteria

1. The intracerebral hemorrhage is caused by secondary factors (e.g. arteriovenous malformation; aneurysm; tumor stroke);
2. Concurrent head injury or history of head injury;
3. Multiple intracranial hemorrhage;
4. Known advanced dementia or disability before ICH happened;
5. With coagulation disorders or history of anticoagulant drugs;
6. With severe liver or kidney dysfunction;
7. With indications of terminal brain hernia (e.g. bilateral mydriasis and loss of light reflex, serious disorders of the vital signs);
8. Pregnancy;
9. With severe intraventricular hemorrhage (CT scan shows that volume of hematoma in the lateral ventricular is over 1/2 of ventricluar), or lateral ventricular enlarge with third and fourth ventricular hematoma.

### Sample size

It was reported that the rate of poor outcome of patients with a hemorrhage volume above 30 ml who received craniotomy is about 49% to 67% [19,21,24]. Previous studies showed that the MIS could decrease the rate of poor outcome by about 7% to 22% [19,21] compared with craniotomy. We assume that the neuroendoscopy and stereotactic aspiration can reduce the rate of poor outcome from 58% to 51%, and a sample size of 2203 patients will be required with a significance level of 5% (2-sided) and a power of 80%. In consideration of ensuring the quality of research, the sample size is enlarged to 2448 (816 In each arm).

### Randomization

Patients will be allocated to neuroendoscopy group, stereotactic aspiration group and craniotomy group by method of simple randomization. The randomized number was created before the trial by SPSS 19.0 and kept in envelopes. The envelopes within randomized number will be sent to the centers before trial begin.

### Blinding

It was impossible to blind either the treating doctors or patients in this trial. In order to ensure the quality of this research, the assessors will be blinded. The outcomes of patients will be measured and filled in paper Case Reports Froms (CRF) by special researchers who would not take part in the allocation and the treatment.

### Treatment

After eligible patients are admitted to the hospital, the investigator will collect the basic information of patients, sign the informed consent and identify the randomized assignment as soon as possible. For all the patients, best medical treatments followed the recommendation of 2010 ASA/AHA intracerebral hemorrhage guidelines and preoperational preparation will be conducted at the same time. The patients will receive the corresponding surgery based on the result of randomization. All the surgeries will be performed as soon as possible after the randomization.

#### Craniotomy

This group of patients will receive hematoma evacuation by craniotomy. Well-trained neurosurgeons will decide surgical approaches according to the result of neuronavigation. The hematomas will be evacuated as much as possible with the help of operation microscope following the principle of “no brain stretch, electrocoagulation with small power, and slight aspiration”. A “Rebleeding test” will be performed after hemostasis. For patients with hypertension, the blood pressure will be slowly elevated to the level before the anesthesia, and for other patients, a raise of 20 ~ 30 mmHg will be gained. The anesthetist will maintain this level of blood pressure for 10 minutes. Only if there is no new bleeding site, neurosurgeons can start to close.

#### Neuroendoscopic surgery

This group of patients will receive hematoma evacuation by endoscopic surgery. Neurosurgeons will decide surgical approaches according to the result of neuronavigation. The hematomas will be evacuated as much as possible with the help of angled endoscope, but the stiffly attached clot won’t be removed by force in order to prevent unnecessary damage, and the restricted intracavity operation will be the principle during the hematoma evacuation. “Rebleeding test” will be conducted before close.

#### Stereotactic aspiration

This group of patients will receive hematoma evacuation by stereotactic aspiration. The puncture site will be based on the result of neuronavigation aimed to avoid functional domains and blood vessels. After puncture needlepoint placed to the center of the hematoma, the needle will be fixed onto the skull. As much of the hematoma as possible will be aspirated. Urokinase will be injected through the puncture needle afterward. The dosage of urokinase is based on the volume of hematoma (10000–50000 U). CT scan will be performed immediately after the procedure.

### Data collection and follow-up

After patient enrollment, we will collect their basic data at neurological evaluation, functional status assessment and imaging for baseline information, and follow up data will be collected at the time of seven days, one month, three months and six months after surgery. The plan of data collection is showed in Table 1. All the data will be recorded in the CRF in time. The filled CRF will be sent to trial secretary expeditiously.

**Table 1:** **Data collection program**

	**Baseline**	**7 days**	**1 months**	**3 months**	**6 months**
**GCS**	√	√			
**GOS**			√	√	√
**NIHSS**	√	√	√	√	√
**mRS**			√	√	√
**BI**			√	√	√
**Laboratory examination**	√	√	√	√	√
**Imaging**	√	√	√		

GCS: Glasgow Coma Scale; GOS: Glasgow Outcome Scale; NIHSS: National Institute of Health stroke scale; mRS: modified Rankin Scale; BI: Barthel Index.

### Outcomes

#### Primary outcome

The primary outcome of this study is the unfavorable outcome at 6 months. The unfavorable outcome includes death and dependency after randomization. The dependency will be defined by using the Glasgow Outcome Scales (GOS) at 6 months after randomization.

#### Secondary outcomes

The secondary outcomes include: mortality at 30 days, and 6 months after surgery; neurological functional status of 6 months after surgery measured by GOS, Modified Rankin Scale (mRS), Barthe index (BI), and National Institutes of Health Stroke Scale (NIHSS) separately; complications include rebleeding, ischemic stroke, intracranial infection; days of hospitalization.

### CT scan

Pre-planned CT scan is at admitting, 1 day, 3 days, and 7 days after operation. Unplanned CT scan is decided by the doctors if necessary. The volume of hematoma is calculated by the formula A*B*C/2. Rebeeding is defined as the postoperative hematoma volume was either greater than which before the operation or there was a less than 5-ml difference between the pre- and postoperative hematoma volume measurement.

### Statistical analysis

The “intension to treat” analysis will be applied in this study. The descriptive analysis of all measurement data is implemented using mean, standard deviation, median, interquartile range, maximum and minimum indicators; the descriptive analysis of count data is implemented using index such as rate, ratio and RR. The primary analysis will be a simple categorical frequency comparison for unfavorable outcome by means of Chi-square test, so will the categorical data of secondary outcomes. The continuous outcomes will be analyzed by t-test. The log-rank method will be used in the analysis of time- to-event type of outcomes. Subgroup analysis stratified by age, location of hematoma, volume of hematoma, GCS at hospitalization, with/without intraventricular hemorrhage, and midline shift is preplanned. Statistical inference is conducted using the above-mentioned hypothesis testing method and confidence interval may also be used when necessary. P values ≤0.05 (α = 0.05) will be considered statistically significant. All statistical analysis is performed using SAS or SPSS statistical software.

### Data and safety monitoring

An independent data and safety monitoring board (DSMB) will monitor the safety and efficacy of this trial. The DSMB consists of neurosurgeons, neurologists, radiologists, statisticians, and data managers. Members of the DSMB will meet in person twice a year to review the trial data. Interim analyses will be conducted. The DSMB will stop the trial if one of the treatments shows advantage or higher incidence of severe adverse effect (SAE) than the other two at a very high significance level (differences of more than 3 SD). The adverse event (AE) is any undesirable syndrome of enrolled patients happens during this study. The SAE is defined as death and persistent vegetative state. Both AEs and SAEs will be recorded in the CRF in detail. If SAE happens, researchers must notify it to the DSMB in 24 hours.

### Study organization

This study is initiated by Department of Neurosurgery in West China Hospital, Sichuan University. West China Hospital is a 4300-bed, superior large comprehensive teaching and researching hospital in China. The Department of Neurosurgery, founded in the 1950s, is one of the earliest neurosurgery specialty wards in China and the largest neurosurgery unit in Southwestern China. A total of 20 centers will participate this study, and the list of centers is showed in the acknowledgement.

## Discussion

SICH has always been a frequently discussed topic in neurology and neurosurgery. Though there are over 10 randomized studies aimed to find the best treatment for patients with sICH, it is still very difficult to select individualized treatment for each patient. MISTICH is the first large-scale randomized, parallel-group clinical trial to provide robust evidence for clinical practice by assessing the safety and efficacy of different minimally invasive surgical methods for sICH. A total of 2448 patients will be enrolled in this study. The primary outcome is unfavorable outcome at 6 months. The complications and trends towards improved neurological outcome will also be investigated in this study.

## Abbreviations

sICH: Spontaneous intracerebral hemorrhage
MIS: Minimally invasive surgery
MISTICH: Minimally invasive surgery treatment for spontaneous supratentorial intracerebral hemorrhage
GCS: Glasgow coma scale
GOS: Glasgow outcome scale
NIHSS: National institute of health stroke scale
mRS: Modified rankin scale
BI: Barthel index
DSMB: Data and safety monitoring board
SAE: Severe adverse effect
AE: Adverse effect
